# Central Auditory Masking by an Illusory Tone

**DOI:** 10.1371/journal.pone.0075822

**Published:** 2013-09-11

**Authors:** Christopher J. Plack, Andrew J. Oxenham, Heather A. Kreft, Robert P. Carlyon

**Affiliations:** 1 School of Psychological Sciences, University of Manchester, Manchester, United Kingdom; 2 Department of Psychology, University of Minnesota, Minneapolis, Minnesota, United States of America; 3 Department of Otolaryngology, University of Minnesota, Minneapolis, Minnesota, United States of America; 4 Medical Research Council Cognition and Brain Sciences Unit, Cambridge, United Kingdom; Hotchkiss Brain Institute, University of Calgary, Canada

## Abstract

Many natural sounds fluctuate over time. The detectability of sounds in a sequence can be reduced by prior stimulation in a process known as forward masking. Forward masking is thought to reflect neural adaptation or neural persistence in the auditory nervous system, but it has been unclear where in the auditory pathway this processing occurs. To address this issue, the present study used a “Huggins pitch” stimulus, the perceptual effects of which depend on central auditory processing. Huggins pitch is an illusory tonal sensation produced when the same noise is presented to the two ears except for a narrow frequency band that is different (decorrelated) between the ears. The pitch sensation depends on the combination of the inputs to the two ears, a process that first occurs at the level of the superior olivary complex in the brainstem. Here it is shown that a Huggins pitch stimulus produces more forward masking in the frequency region of the decorrelation than a noise stimulus identical to the Huggins-pitch stimulus except with perfect correlation between the ears. This stimulus has a peripheral neural representation that is identical to that of the Huggins-pitch stimulus. The results show that processing in, or central to, the superior olivary complex can contribute to forward masking in human listeners.

## Introduction

Auditory sensitivity is reduced directly after the termination of a sound. This phenomenon, known as “forward masking,” has important consequences for the perception of speech, music, and environmental sounds, all of which fluctuate over time. For example, when we are having a conversation in a noisy room we can make use of dips in the fluctuating background noise to better “hear out” the talker of interest [1,2]. Our ability to do so depends on how the forward masking produced by preceding peaks in the background persists over time.

It is thought that forward masking depends on either neural *adaptation*, in which the neural response is suppressed after stimulation by the masker, or neural *persistence*, in which the neural response to the masker continues after stimulation, swamping the response to the subsequent signal [3,4]. However, it is unclear where in the auditory nervous system forward masking occurs. After transduction in the cochlea, the neural signal is carried by the auditory nerve to the cochlear nucleus (CN), the superior olivary complex (SOC), the nuclei of the lateral lemniscus, and the inferior colliculus (IC), before being transmitted to the medial geniculate body in the thalamus and then finally to the auditory cortex. Single-unit neurophysiological measures in non-human mammals suggest that adaptation in the auditory nerve or CN is not sufficient to account for psychophysical forward masking [5,6], but by the level of the IC [7] and auditory cortex [8] adaptation or post-stimulus suppression may be sufficient to account for the perceptual results. However, there are no comparable findings in human listeners, and it is unclear whether the neurophysiological results relate directly to perceptual performance. A knowledge of the neural loci at which forward masking occurs would be of practical as well as theoretical importance, given recent developments in auditory prostheses that bypass the auditory nerve and restore hearing via electrical stimulation of the CN or IC [9,10].

**Figure 1:**
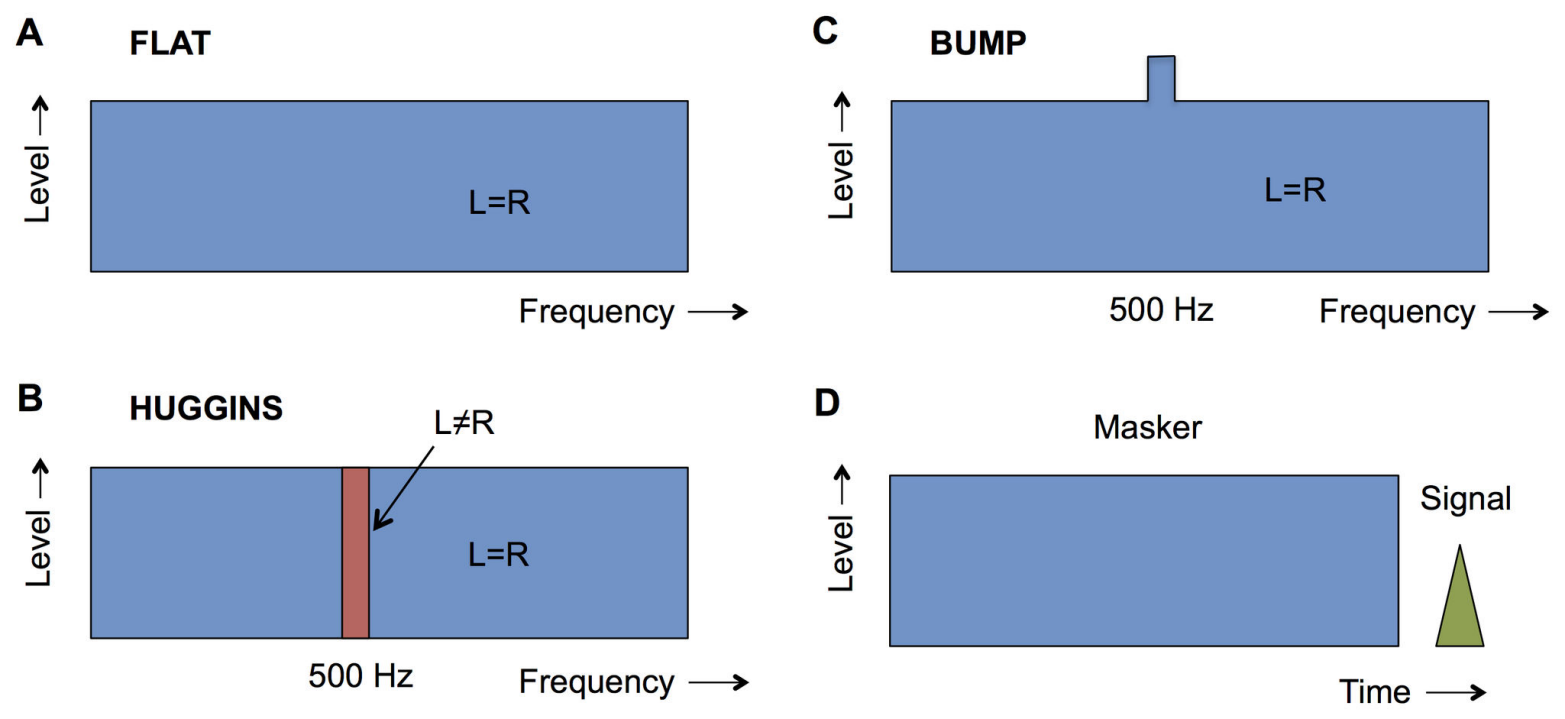
Stimuli. Schematic illustrations of the spectra of the masking noise for the Flat (A), Huggins (B), and Bump (C) conditions. “L” and “R” refer to left and right ears respectively. The decorrelated band (L≠R) for the Huggins stimulus is shown in red. D, A schematic illustration of the temporal characteristics of the stimuli.

Huggins pitch is an illusory tonal sensation that depends upon stimulation to both ears (“binaural” stimulation) [11,12]. It is created by presenting a wideband noise, which is identical in the two ears except for a narrow frequency band that differs (i.e., is *decorrelated*) between the two ears (Figure 1B). Listeners hear a musical pitch with a frequency roughly equal to the center frequency of the band. However, the input to each ear is just a noise with no tonal quality. Only when the inputs to the ears are combined by the central auditory system does the pitch sensation emerge from the decorrelated band. This combination of information is thought to occur in the SOC [13], and is part of the processing that allows us to “separate out” sounds from different directions by virtue of their different times of arrival at the two ears [14].

The present experiment was designed to answer two questions. First, does forward masking depend in part on auditory mechanisms central to the combination of information from the two ears? Second, does the neural processing that produces the Huggins-pitch sensation involve an enhancement in the activity of neurons tuned to the decorrelated band? To address these questions in humans, a non-invasive behavioral method was used. Forward masking by a Huggins pitch stimulus was compared to that by a noise perfectly correlated between the two ears.

## Materials and Methods

### Stimuli

Three masker conditions were tested in the main experiment (Figure 1). The reference condition (“Flat”) was a Gaussian noise, low-pass filtered at 1200 Hz, and with a spectrum level of 50 dB SPL. The same noise was presented to both ears (Figure 1A). The Huggins pitch stimulus (“Huggins”) was similar to the Flat condition except that a **π** phase shift was introduced between the ears over a rectangular frequency band from 475 to 525 Hz (Figure 1B). The final condition (“Bump”) was similar to Flat except that the spectrum level within the rectangular band between 475 to 525 Hz was increased in level by 7 dB, relative to the spectrum level in the remainder of the noise (Figure 1C). As for the Flat condition, the Bump stimulus was identical at the two ears. Previous studies have shown that an increase in level of a narrowband portion of about 7 dB within a wideband noise produces a pitch salience similar to that of a Huggins-pitch stimulus [15,16]. Each masker had a total duration of 400 ms, including 10-ms raised-cosine onset and offset ramps. Independent noises were generated for each presentation.

The signal was a 500- or 900-Hz pure tone identical in the two ears and presented after the masker (Figure 1D). The 900-Hz condition was included to control for the possibility that, in the Huggins condition, the perception of a tone in the masker raised thresholds via a non-sensory effect, such as cognitive “distraction”, rather than the frequency-specific sensory mechanisms believed to be responsible for forward masking. The duration of the signal was 20 ms including 10-ms raised-cosine onset and offset ramps. The silent interval between the offset of the masker and the onset of the signal was 10 ms. Stimuli were generated digitally and were output by a 24-bit soundcard (Lynx22, Lynx Studio Technology, Costa Mesa, CA) set at a clocking rate of 48 kHz. The headphone output of the soundcard was fed via a headphone buffer (HB6, Tucker-Davis Technologies, Alachua, FL) and headphones (HD580, Sennheiser, Old Lyme, CT) to listeners, who were seated in a double-walled sound-attenuating chamber.

A second experiment, conducted after the main experiment, used maskers that covered the same frequency range as the decorrelated band in the Huggins condition from the main experiment. In other words, each masker consisted of a 400-ms Gaussian noise, which was filtered from 475 to 525 Hz by setting the amplitudes of the spectral components outside the passband to zero. The noise was either identical in the two ears (N_0_S_0_) or had a **π** phase shift between the ears (N_π_S_0_). The spectrum level of the noise was again 50 dB SPL. An additional noise, filtered from 1500 to 6000 Hz with a spectrum level of 30 dB SPL and the same binaural relationship as the masker, was gated with the masker to reduce the "confusion" effects that can occur when a pure tone signal is presented after a narrowband masker [17]. The 500-Hz signal and the masker-signal interval were the same as those in the main experiment.

### Procedure

On each trial, listeners were presented with three observation intervals separated by 500 ms. Two intervals contained the masker only; one interval (chosen at random with uniform probability) contained the masker plus the signal. Listeners were required to select the interval containing the signal (three-alternative forced choice). The level of the signal was varied between trials using a “two-down one-up” adaptive staircase to find the level at which the signal was just masked according to a 71% detection criterion [18]. The level of the signal was decreased by the step size after every two consecutive correct responses, and increased by the step size after every incorrect response. The step size was 4 dB for the first 4 “turnpoints” (transitions between ascending and descending level) and 2 dB thereafter. In each block of trials, 10 turnpoints were measured and the threshold estimate was taken as the mean level at the last six turnpoints. Six such estimates were made for each listener, and the results of the last five threshold estimates were averaged. In the main experiment, the six conditions (three masker conditions and two signal frequencies) were presented in a pseudo-random order for each listener and each repetition of the conditions. In the second experiment, the two masker conditions were presented in a pseudo-random order for each listener and each repetition of the conditions. In addition, four threshold estimates were made for each signal frequency in the absence of the masker.

Six listeners were tested in the main experiment, and seven in the second experiment. One of the listeners (L4) was tested in both experiments. All listeners had normal hearing. Listeners were tested individually and responded to the stimuli via a computer keyboard. A display on the computer monitor indicated the time of occurrence of the observation intervals and provided feedback as to whether the response was correct or incorrect. All listeners provided written informed consent. The experimental protocol was approved by the Institutional Review Board of the University of Minnesota.

## Results

**Figure 2:**
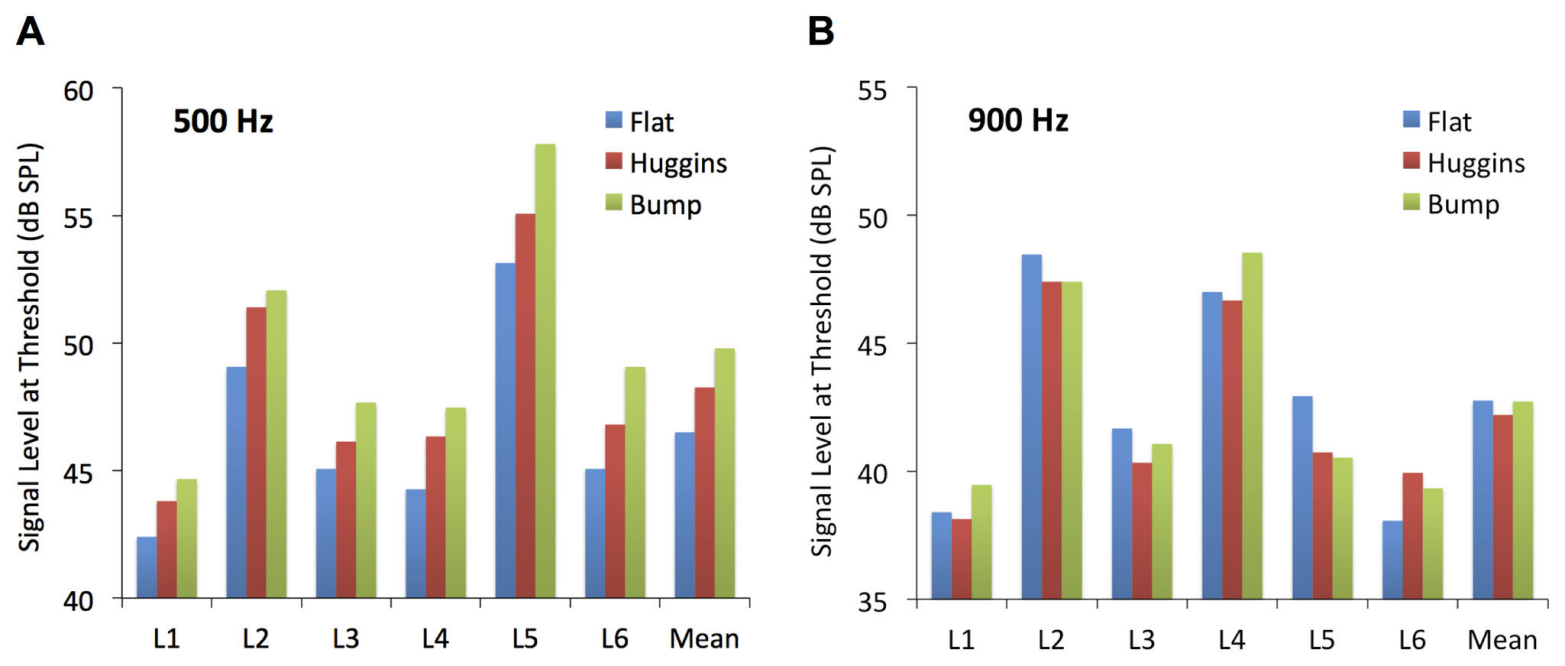
The individual and mean results of the main experiment. The different panels show the results for the 500 Hz (**A**) and 900 Hz (**B**) signal frequencies. Signal levels at threshold for the Flat, Huggins, and Bump conditions are shown by the blue, red, and green bars respectively.

The mean thresholds for the pure-tone signals in quiet were 19.6 dB SPL (standard error, SE, = 1.9 dB) and 16.3 dB SPL (SE = 1.2 dB) at 500 and 900 Hz respectively. The results of the main experiment are shown in Figure 2. For the 500-Hz signal (Figure 2A) the same pattern is apparent for each of the six listeners. Masked threshold (and hence amount of masking) increases from Flat to Huggins to Bump. The mean difference in threshold between Huggins and Flat is 1.8 dB (SE = 0.2), and the mean difference between Bump and Huggins is 1.5 dB (SE = 0.3). A one-way related-means analysis of variance (ANOVA) revealed a highly significant effect of masker condition [F(2,10) = 58.07, p < 0.001]. Individual t-tests (Bonferroni corrected) showed that each masker condition differs significantly from the others (p < 0.02 in each case).

For the 900-Hz signal (Figure 2B) the results are variable across listeners, and there is no consistent effect of masker condition. A one-way ANOVA revealed no significant effect of masker condition [F(2,10)=0.67, p=0.54)]. A two-way ANOVA, considering just the Flat and Huggins conditions at each frequency, revealed a significant interaction between masker condition and frequency [F(1,5) = 14.69, p = 0.012], reflecting the finding that the mean difference between Flat and Huggins is greater at 500 Hz than at 900 Hz.

**Figure 3:**
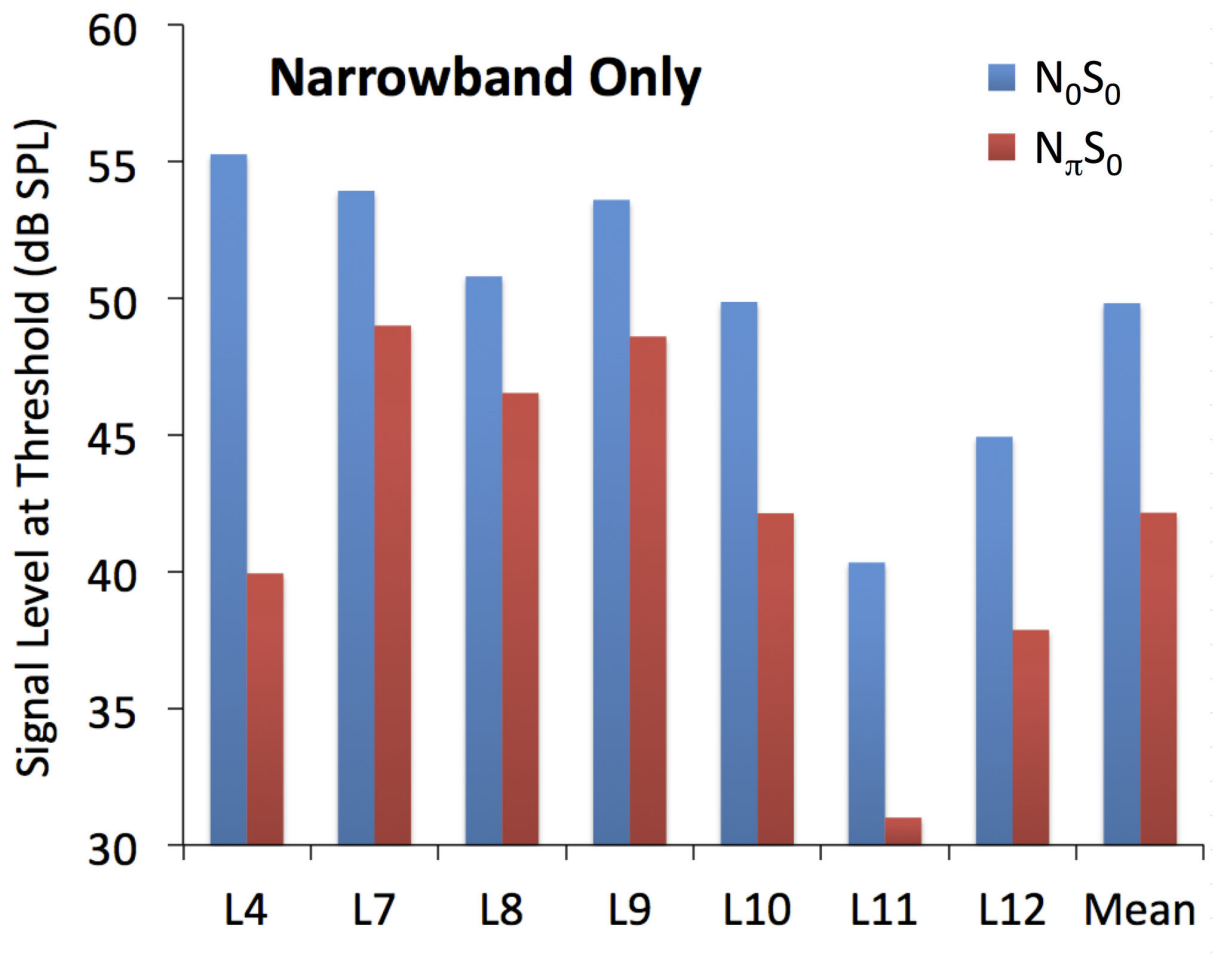
The individual and mean results of the second experiment. Signal thresholds for the N_0_S_0_ and N_π_S_0_ conditions are shown by the blue and red bars respectively.

The results of the second experiment are shown in Figure 3. For each listener, the N_0_S_0_ condition produced more masking than the N_π_S_0_ condition. The mean difference in masked threshold is 7.7 dB, and a paired t-test revealed that this difference is significant (p = 0.0018). In other words, the narrowband noise produced significantly more masking when it was identical in the two ears than when it was different in the two ears. This is opposite to the finding in the main experiment, in which *less* masking was observed in the Flat condition (in which the noise was identical between the two ears) than in the Huggins condition (in which the narrowband noise was different between the ears).

## Discussion

### Forward masking by a Huggins pitch stimulus

The results show a difference in masking between the Flat and Huggins conditions at 500 Hz. This demonstrates that a contribution to forward masking must arise from processes central to the combination of the inputs from the two ears, i.e., in the SOC or later. Although the central masking effect is small (1.8 dB), it is a substantial portion of the masking effect for the Bump stimulus (3.3 dB), which has a similar pitch salience. The lack of an effect of masker condition for the 900-Hz signal suggests that the additional masking was specific to the frequency region of the decorrelated band or spectral bump. This is consistent with the frequency selectivity of the peripheral auditory system, and implies that the additional masking was not a consequence of a general cognitive “distraction” produced by the perception of a tone in the masker. It is possible that masking of the 500-Hz signal by the Huggins stimulus was influenced by “confusion” effects that are sometimes observed in forward masking, such that it was harder to differentiate the masker from the signal as they both produced a tonal sensation. However, confusion effects are not thought to occur when the overall masker bandwidth is wide [17], as was the case here, as this provides an across-frequency cue for differentiating the masker from the signal.

Forward masking has been observed previously between a masker and a signal presented to opposite ears. This would appear to imply an interaction between the masker and signal after the two have been combined in the SOC. However, the masking is much less than when masker and signal are in the same ear, and only occurs at short masker-signal intervals (typically less than 10 ms) [19]. This contralateral masking could be a consequence, at least in part, of the neural feedback loop that provides efferent gain reduction to the ipsilateral and contralateral cochleae [20,21]. Any gain reduction would reduce the sensitivity to the subsequent signal, elevating threshold. This processing, although perceptually significant, is distinct from the mechanisms that are mainly responsible for forward masking.

It is well established that interactions between the two ears can affect masked thresholds. For example, if a tonal signal is identical in the two ears but a simultaneous noise masker is inverted between the two ears (N_π_S_0_), the signal is easier to detect than if the masker is identical in the two ears (N_0_S_0_) [22]. A release from masking is also observed if the noise is identical between the two ears but the signal is inverted between the two ears (N_0_S_π_). The difference in masked threshold when the masker and signal have different binaural relations is called the “binaural masking level difference” (BMLD). The BMLD has also been demonstrated for forward masking conditions [23–26], providing further evidence for a central influence on forward masking. However, a substantial forward-masking BMLD usually occurs only for low-frequency signals and short masker-signal intervals (< 20 ms). Hence, the results could be affected by simultaneous interactions between the masker and signal, due to “filter ringing” – persistence of mechanical vibrations in the cochlea after the offset of sound [27,28], which has been shown to have perceptual consequences in monaural conditions [29–31]. In other words, although the *release from masking* for the inverted signal may be due to central factors, this does not imply that the processes that produce the masking are central. In contrast, the results of the main experiment here provide a clear demonstration that psychophysical forward masking involves central processes.

The results of the main experiment are effectively a “negative” forward-masking BMLD, as when a narrowband portion of the masker was different between the two ears (the Huggins condition; effectively N_π_S_0_) it produced more, not less, masking. The contrast is highlighted by the results of the second experiment. When the surrounding masking noise was removed, the decorrelated band produced considerably *less* forward masking than the band that was identical between the two ears. This is a forward-masking BMLD, comparable to the results of previous forward-masking BMLD studies [23–26].

The Bump stimulus produced more masking than the Huggins stimulus. If it is assumed that the *central* representations of these two stimuli are similar, based on the fact that they both produce a similar level of pitch salience [15,16], the difference in masking between Huggins and Bump may reflect the contribution to forward masking from processes prior to binaural integration, since the Bump stimulus has more energy at 500 Hz, and hence a stronger peripheral representation, than either the Flat or Huggins stimuli. The fact that the difference between Huggins and Bump is similar to the difference between Flat and Huggins suggests that peripheral and central processes may contribute to forward masking to similar degrees.

The present finding that central mechanisms contribute to forward masking is consistent with neurophysiological measures of adaptation, or post-stimulus response suppression, at various levels in the auditory pathway of non-human mammals. Analyses based on signal detection theory, which estimate the detectability of the signal in relation to the background neural noise, suggest that adaptation at the level of the auditory nerve or CN is insufficient to account for behavioral thresholds [5,6]. However, estimates of detection thresholds based on post-stimulus suppression in some IC neurons are comparable to those measured behaviorally, and show similar behavior with respect to masker level and masker-signal interval [7]. Forward masking thresholds based on post-stimulus suppression of cortical neurons are greater still, and may even exceed behavioral thresholds [8].

### Increased forward masking by Huggins pitch reveals a neural enhancement of the decorrelated band

The finding of increased masking by a Huggins-pitch stimulus compared to a flat noise suggests that the neural representation of the decorrelated band is enhanced relative to that of a correlated band, in a way that is *equivalent to* an increase in physical stimulus level (as exemplified by the Bump stimulus). The results of the second experiment demonstrate that this relative enhancement is dependent on the presence of background noise outside the decorrelated band. When the background noise was removed, the decorrelated band produced *less* masking than the correlated band. In other words, it is the contrast between the decorrelated band and its surrounds that produces the enhancement, not the decorrelation itself.

Some evidence for neural enhancement can be found in measures of the human cortical response to Huggins-pitch stimuli. The human neuromagnetic evoked response shows an enhanced response to Huggins-pitch sounds when preceded by a matched noise background identical in the two ears, and equivalent to the Flat stimulus used here [32,33]. This “pitch onset response” (POR) has a latency of 150-200 ms, and has been localized to a region close to primary auditory cortex (PAC). Furthermore, the Huggins POR is similar to that for a pure tone in noise with matched salience [33]. The neuromagnetic results are therefore broadly consistent with the results of the present experiment in suggesting a similar representation for a decorrelated Huggins band and for a tone or narrowband noise with a “monaural” pitch of similar salience. Human functional magnetic resonance imaging studies also show that regions adjacent to PAC, in lateral Heschl’s gyrus or anterior planum temporale, produce an increase in activity to a Huggins stimulus compared to a matched noise identical in the two ears [34,35].

In the “equalization-cancellation (EC)” model of binaural processing [36] the signals at the two ears are subtracted, causing a cancellation of the energy in frequency regions that are correlated between the ears. This would result in a Huggins pitch, not by boosting the frequency region corresponding to the narrow decorrelated band, but by attenuating the background [12,37]. According to the EC model, the difference in forward-masked thresholds between the Flat and Huggins conditions could be explained if the correlated Flat noise were partially cancelled across all frequencies, including the on-frequency band. However, this does not explain easily why threshold in the Bump condition is higher than that in the Huggins condition, since the noise is fully correlated in the Bump condition. In addition the signal itself would be cancelled by this mechanism, as it has the same interaural delay (zero) as the correlated noise. Hence this mechanism is an unlikely candidate for an optimum signal detection strategy. Instead, masking in the Bump condition may depend more on monaural processes. Alternatively, in the physiologically based cross-correlation models of Jeffress [38] and Colburn [39,40], for coincidence detectors selective to the interaural delay of the signal (zero) the response to the correlated background is enhanced relative to the response to the decorrelated band. Hence this model also cannot account easily for the current findings.

Both the EC and cross-correlation models can predict the increase in detectability of a signal when it is presented in a different binaural relation relative to a masking noise (the BMLD). The results of the present main experiment are intriguing as the Huggins condition is a BMLD condition in which presenting the noise and the signal with different binaural relations produces *more* (not less) masking.

### Summary and implications

Our results provide behavioral evidence that a contribution to forward masking can arise from binaural processing at the level of the SOC/IC and beyond. They suggest that the response to an interaurally decorrelated band of noise is enhanced in the auditory system, in a way that affects perception and is not under conscious control.
